# Odontalgia

**Published:** 1895-03

**Authors:** N. S. Hoff

**Affiliations:** Ann Arbor, Mich.


THE DENTAL REGISTER.

VOL. XLIX.] MARCH, 1895. [No. 3.

### COMMUNICATIONS.

### Odontalgia.

BY N. S. HOFF, D.D.S., ANN ARBOR, MICH.

Read before the Ohio State Dental Society, December, 1894.

Several years ago, when I was younger in professional life, an older practitioner proposed as a subject for consideration by our local dental society, "What would you do with a case of toothache?" I was surprised and somewhat indignant that he should refer to this malady in such indefinite and unscientific terms, and especially to invite a society of professional gentlemen to discuss it.

During the past two months I have apparently lived in an atmosphere of toothache. It has seemed to me there was no disease so common, or one which presents so many vexing problems, or brought me so many patients. Living in a college town, I have had toothaches from almost every State and territory in the Union, Canada, Germany and England, presenting almost as many varieties as patients, and when your executive committee invited me to write a paper for the meeting and refused to assign me to a subject, I could not get away from toothache, and shall take pleasure in unburdening my mind even if I do bore you.

It is exceedingly difficult to define odontalgia satisfactorily and comprehensively, without qualifications. In the mind of the laymen it covers all diseases of the teeth and associated parts, especially when painful. But the dental practitioner will associate the disease with numerous functional disturbances of the dental organs and tissues. The word toothache to him is comprehensive but not definite.

In the *American Journal of Dental Science* for July, 1853, I find an article on odontalgia by J. P. Fogg, M.D., from which I shall quote the opening paragraph for the graphic and forcible way in which it describes the malady, and for its historic interest and as an introductory to my theme.

"The simple definition of the technical term odontalgia is to be found in our vernacular word toothache. No theory whatever is implied in it. It simply means what it emphaticaly is, *a pain in a tooth.* The character of this pain is protean. It may be a slight uneasiness, it may be an intolerable anguish. It may be dull, heavy, constant like rheumatism, acute and lancinating, paroxysmal, darting, boring, throbbing. Any term of the frightful vocabulary of human suffering may be applicable to this torture. It may be cumulative, beginning moderately and increasing to intense severity, or it may at once attack the sufferer, armed in all its terrors. Yet toothache is, after all, only a symptom of some lesion, either in the pulp, the tooth, the jaw, the nerve, the brain, or the system at large."

No authoritative classification of the different phases of odontalgia will admit of a brief and comprehensive consideration, such as may be necessary to properly present the subject at this time, and I take the liberty of condensing the accepted classification and considering it under five headings simply for convenience.

First: Exposure of the dentine by accident or caries.

Second: Exposure and disease of the pulp.

Third: Disease of the peridental tissues.

Fourth: New growths in the pulp tissue or upon the roots.

Fifth: Reflex irritation.

Just why exposed dentine causes pain in the tooth we do not at present so clearly understand, as that it does. Clinical experience has practically established the fact of a wide difference in the susceptibility of dentine, when exposed in different patients, to pain. Very slight exposures will cause pain in some persons, while extensive exposures in others will cause little or no pain. It is also true that at different periods exposed dentine is more likely to cause pain in the same persons, and accidentally different teeth in the same mouth may manifest differences in the degree of sensibility. The old writers accounted for pain or sensation in the dentine on the theroy that it was subject to inflammatory processes. But on account of the absence of the essential elements of inflammation this theory is not now believed. Some authors think the dentine is endowed with sensory nerve fibers, although they confess their inability to demonstrate their existence, or to trace any connection between the nerves of the pulp and the dental fiber or organic tissue of the dentine. Bodecker, in his recent work, Anatomy and Pathology of the Teeth, page 272, says: "It is impossible to admit of the existence of a connective tissue holding nerves alone in its constiuent soft parts. Neither have we, nor has Retzius in his recent investigations, been able to trace a direct inosculation of the dentinal fibrillæ with the axis fibrillæ of the nerves so abundantly distributed throughout the pulp tissue. As soon, however, as we admit that the dentinal fibrillæ are formations of living matter, the same as are the nerves, all difficulties vanish in explaining the transmission of sensation from the periphery of the dentine to the nerves of the pulp tissue. Living matter is contractile. Nerves are made up of living matter, and owing to their reticulated or beaded structure are fittest for that transmission of contractions from the periphery to the nervous centers which we call sensation. Contraction of the dentinal fibers transmitted into the reticulum of protoplasm at the periphery of the pulp, and thence into the ultimate nerve fibrillæ—all of which formations are proved to be continuous—is sufficient to explain the transmission of sensation, or speaking bluntly, of pain."

This theory, whether we can accept it entirely or not, is as satisfactory as any other and has much to cause us to accept it. It is plausible and harmonizes with clinical experience and manifestations. The application of agents capable of producing contraction in protoplasm and organized tissue elsewhere, we find produce pain when applied to exposed dentine, such as cold, excessive heat, escharotics, caustics, and other chemical and mechanical irritants. All successful treatment of exposed dentine depends upon its being protected mechanically or by narcotic and anæsthetic drugs, from the irritation of these agents, or upon the disorganization of the organic tissue in the dentine so that irritants produce no impression when applied. It, therefore, seems clear that the theory is worthy of confidence, and all principles for treatment of sensative dentine and from the relief of pain from exposure of the dentine should be based upon it until some one demonstrates its error or gives us a more plausible one. The technical resources for the cure of toothache from this cause are so various that any attempt to detail them here would be a burden. We rest our practice on the acceptance of the contractile theory, leaving to individual cases the selection of procedure and remedy.

The second division of our subject, Exposure and diseases of the pulp, presents a different and more complicated series of conditions, requiring prompt and definite treatment, as the symptoms are frequently manifested with great energy, because of the peculiar environment of the pulp and its sensative nature. The pulp is richly endowed with blood vessels and nerves contained in a matrix of connective tissue. There are lymphatic canals and possibly lymphatic glands. There are few arteries and veins, and these are near the center of the pulp, but the entire organ is richly supplied with blood capillaries, especially toward the periphery. The odontoblast layer on the periphery of the pulp is not highly sensative, but the granular layer immediately beneath contain the nerve endings of the pulp nerves and is highly sensitive. Irritation of the exposed pulp is followed by pain, depending on the character of the irritant as to its penetrating power and the severity of its action. Mechanical and galvanic shocks cause prompt and severe pain; and because of the favorable structure of the pulp the shock is quickly experienced, not only in the pulp, but in the general sensorium as well. Chemical irritants act more slowly, depending on their relative solubility. The power of their impression will depend upon their characteristic physiological or chemical affinities. The pain produced in each case will correspond with the time and intensity of the irritation. Irritation of the pulp is always followed by disturbance of its normal circulation. The first effect is to produce contraction of the tissues and more or less anemia, which is quickly followed by relaxation and a determination of blood to the part, congestion and incipient inflammation. This condition is scarcely pathological, and yet it requires very nice manipulation, and the proper selection of remedies to prevent serious inflammatory conditions following. Prompt removal of all irritants and provision for complete seclusion, accompanied by the application of local vascular and nerve sedatives. Disinfection with non-cauterant germicide, the application of an antiseptic dressing and the careful adaptation of a protecting filling, are indicated for the relief of the pain and its subsequent recurrence.

*Inflammation of the pulp*, results from inoculation under conditions of general systemic infirmity, or because of excessive exposure, and irritation in consequence of mechanical, chemical or physical excitants. There are the usual symptoms of irritation and in addition the entire pulp takes on a persistent congested condition which in a measure suspends the normal function of the pulp. There is pain, not only of the acute variety, but the tissues themselves take on a kind of lameness or hypersensitive condition, so that every pulsation of the heart is distinctly felt, causing intense and prolonged suffering. This condition is not amenable to treatment with the ordinary local remedies used for pulp irritation, at least not for curative results. The results of this affection are somewhat varied and the prognosis is uncertain. Usually it means the utter annihilation of the pulp as a vital organ. In favorable cases: good systemic health, youth and the absence of destructive infection, the chances of successful conservative treatment are correspondingly better. In many cases it is practicable to save the pulp even after extensive inflammation. It is of the utmost importance that the actual condition present should be carefully determined, in order that needed and useful pulps be not sacrificed indiscriminately on principle. And also to prevent needless suffering for the patient in a futile attempt to save pulps which ought to be subject to radical treatment at once.

The treatment of this condition for the relief from pain temporarily, can in almost every case be successfully accomplished by the removal, as far as practicable, of all local irritants; the application of astringent, non-irritating sedative; an antiseptic dressing to be covered with such material as the case will indicate, to prevent further irritation and the dissipation of the dressing by solution in the saliva. Counter stimulation of some of the systemic eliminating organs to withdraw the excess of fluid from the pulp, such as a saline cathartic. If this is not advisable a heart sedative will give temporary relief and enable the tissues of the pulp to recuperate and secure a more normal circulation and function. After the inflammatory condition has subsided, the pulp may be covered with a suitable non-irritant filling material and the carious cavity filled with a more resistant material, one that may be removed without applying excessive force in case of a reappearance of the difficulty. The conservative treatment of pulps so diseased will necessarily depend so largely upon individual conditions, that it would be inpracticable to even suggest a treatment for the varied conditions which must be met. The condition of the pulp itself, as to the character and extent of the disease; the personal idosyncrasies of the patient as to general health; the difficulties of exact technical manipulation; the importance of the individual tooth and the subsequent service required of it, are to be taken into consideration. Placing crowns, bridge attachments, regulating appliances, or requiring constant and heavy service in any way, will be likely to thwart all efforts for the conservative treatment of teeth having had inflamed pulps. If any such service is to be required of a tooth soon after treatment, it will be better to destroy the pulp at once and avoid almost certain secondary inflammation. If the indications are that there is extensive inflammation of the pulp and it is desirable to insert cement or amalgam fillings, or even gold fillings, it will save much inconvenience and suffering to destroy and remove the pulp at once.

The third stage in-exposed and diseased pulp is what is sometimes called gangrene of the pulp. We may have gangrene of the entire pulp or only of the exposed surface. Where the exposed portion of the pulp only is involved, it takes the form of a slough, with little or no pain except when irritated. This condition is not always the cause of severe toothache, but is sufficiently troublesome to require attention. The indications usually are for entire destruction of the pulp, but a few conservative practitioners recommend excising the diseased portion, and by palliative treatment save the remaining portion. In my own hands this procedure has never been successful in permanent results. The most painful form of pulp gangrene occurs when the pulp is inclosed in the pulp chambers by fillings or debris of caries. The generation of gas in the putrefactive process creates pressure and consequent irritation of the nerves of the pulp and may extend to the periosteum. The pain is continued, severe and somewhat vascillating, owing to the thermal changes experienced through the tooth. The pulp is highly congested and if the cause of the irritation be not removed inanition and dissolution follow. Conservative treatment of such pulps is not advisable, but the excessive pain can be reduced by making an opening directly into the pulp chamber to allow the gas generated in the putrefactive process to escape. After which the pulp can be anæsthetized and removed entirely. The root canal should then be sterilized and filled and also the carious cavity of the tooth. It is sometimes difficult to locate the affected tooth as there may be no complication with the peridental membrane, and because of reflex irritation the pain will be manifested in another tooth, or in associated organs, the eye, ear and face. Testing suspected teeth with extreme heat or cold will result in exaggerated symptoms when the affected tooth is tested. The heat serving to expand the gas which results from putrefaction and as it can not escape from the pulp chamber it makes pressure on the inflamed tissues and consequently pain. In the same tooth the application of cold will cause pain at first by shock, but immediately relief will come because of condensation of gas and relief of pressure, and also because of the fact that contraction of the tissues by shock forces the fluid out of the pulp chamber, and restores the tone to the blood vessels. If suppuration has not yet set in, and still there is excessive inflammation, the application of cold will produce pain, not only by shock, but by its stimulating effect through the vasso-motor system upon the collateral blood circulation, throwing more blood into an already congested pulp which has no room for expansion. The use of heat in such cases will relieve by lowering the blood pressure in the collateral circulation and consequently depleting the pulp. It is evident to everybody, especially one who has tried these agents for this purpose, that great care should be exercised in their use, especially to confine them as much as practicable to a single tooth at a time. But careful use of them will result in the most accurate information. Percussion may aid some, but has no special value unless there be peridental inflammation. Putrefactive changes may be determined by difference in resonance. Reflected light will often be of service in noting the difference in opacity. The history of the case will frequently suggest much that will be of value. An accurate diagnosis will suggest the treatment without great difficulty. As an expedient extracting the offending tooth, this would be a radical measure indeed. Conservative treatment would in such cases be along the line of saving the tooth without the pulp as this organ in this condition is entirely beyond any medical treatment. The first step will be to gain access to the pulp in order to effect its removal. Sometimes it is impracticable to do this because of circumstances; proper instruments are not available, or the patient's health will not admit of so painful an operation as drilling into the canal through a dense tooth or hard filling. General or systemic remedies will be indicated in such emergencies, systemic narcotics, combined with drugs which will depress the heart's action directly, or lower the blood pressure by acting upon the vascular system, or depleting the blood with diaphoretics, cathartics or diuretics. Dover's powder as a narcotic and diaphoretic will answer generally together with a warm foot bath, a reclining position, and in aggravated cases a heart sedative, such as aconite. A warm poultice applied over the region of the affected tooth will relieve congestion and prevent pericemental irritation. If it is possible to secure free access to the pulp at once, the treatment is much simplified. The disorganized tissue should be thoroughly cleared away by syringing the cavity with warm water, the rubber dam put in place to isolate the tooth, and pulp injected with a solution of cocaine and thoroughly removed. If there has been no suppuration to infect the dentine or peridental membrane, the tooth may be sterilized and filled at once. If gangrene has taken place it is better to make a dressing of a disinfectant and defer filling the canal to a subsequent sitting.

The fourth condition of exposed and diseased pulps is a somewhat rare one. *Tumefaction*, except in rare instances, is merely hyperplesia, an excessive growth of the basis substance, or the growth of the basis substance of the pulp until it protrudes from the exposed orifice of the pulp chamber. This condition is not painful except when specially irritated, and the season of pain is not prolonged. The nerve supply is scant but the blood supply is abundant. It is simply a pathological tissue and is incapable of being restored to its normal condition and function. Its destruction is always indicated. This can best be done by anæsthetizing it with cocaine and removing by an operation. Cauterizing chemicals produce intense pain in it and can not be used. Actual and galvanic cautery may be employed, but they are more or less painful and the manipulation of such cauterants is difficult.

Toothache from disease of the peridental membrane is not uncommon. It frequently has an intimate relation to diseases of the pulp, already considered. In fact the majority of diseases of the peridental membrane which cause toothache are the result of destructive inflammation of the pulp. This organ is easily inoculated from a diseased pulp, because of the intimate relationship of the two organs. Its environment is similar to that of the pulp, its nerve supply is not so rich, but it has a very liberal blood supply. When congested by inflammation it thickens and lifts the tooth in its socket, so that unusual pressure and percussion from the occlusion of the teeth serve to exaggerate the already irritated tissue and consequently it becomes exceedingly painful. If the affection is mild in type and acute, it may not cause great inconvenience, but is exceedingly painful when struck. It is sometimes produced by mechanical and external irritants, such as blows, over-malleting, regulating, wedging, application of clamps, etc. In such cases recovery will be prompt after the removal of the cause of irritation. If the cause be infection from an inflamed or suppurating pulp, or the forcing of the debris of a putrid pulp through the apical foramen of the root, or the continued action of irritant or caustic chemicals, a serious inflammatory result may appear, varying in all degrees from irritation to suppuration and infection of the contiguous tissues of the jaw and face and is correspondingly painful. In the beginning of inflammation of the peridental membrane the tooth elongates because of congestion, pressure on the tooth produces pain, as the inflammatory process succeeds the pain increases and changes from tenderness to acute pain and then to a throbbing or jumping character. There seems to be a more powerful heart beat, and if the patient exert himself the rapidity of the action is increased. If suppuration takes place there is general pyrexia and nervousness, and the patient is exceedingly uncomfortable.

The treatment of incipient and acute pericementitis due to inflammatory action set up because of its association with an inflamed pulp, would of course depend upon the treatment of the pulp. The removal of the irritated pulp and the local application of counter irritation will be sufficient to cause it to subside. But when the peridental membrane itself has become affected by inoculation and a destructive inflammatory process has been set up in it a more difficult disease for treatment is encountered. Sometimes its course may be aborted by the continuous local application of counter-irritating blisters, and systemic depletion, disinfection, saline cathartics and antipyretics. Too often the disease must run its course to suppuration. When this is the case all possible means for hastening the process should be employed. The most effective being poultices of various substance to keep the parts warm and moist, thus assisting in the production of putrefactive agencies and the relaxation of the resistance power of the tissues molecularly. So that the pus shall be formed quickly in sufficient quantity to burrow its way through the tissues toward the surface, when by the use of the lance it may be drawn off and with it much of the active agency causing the inflammatory condition. The releasing of the pus will usually give immediate relief. Little further medication is indicated, except to keep the sinus made by the pus open and clean. This should be done with warm water containing some active germicide in solution, until resolution has taken place. If the abscess has been of long standing and there be necrosis of the bone, its removal is indicated as is also the use of local stimulating remedies.

*Calcific growths* in the pulp chamber and on the roots of the teeth are a more frequent cause of pain than we realize. The symptoms are not so markedly manifested as in inflammation of the pulp or peridental membrane. The pain is not severe nor acute, but is more continuous. Calcific deposits in the maxtrix of the tooth pulp are due to the irritation of the pulp through exposed dentine or cementum from caries, abrasion or recession of the gums. No inflammatory process takes place in the pulp, but an abnormal functional excitement, due to this external irritation through the denuded dentine. When these deposits form in the connective tissue of the pulp or on the walls of the dentine, no excessive pain is experienced. But it frequently happens that owing to a hyperæmic condition of the pulp, these formations take place in the capillaries or in the nerve tissues, producing considerable pain. The pain in such cases may be slight in the tooth itself, but by irritation of the reflexes pain is manifested in other, even somewhat remotely situated tissues, such as the eye, ear, the shoulder, the sides of the body, etc., but generally facial neuralgia, which is difficult to locate, as the teeth and muscles of the face and throat are all more or less implicated. The diagnosis is difficult, as there are no apparent local symptoms. Teeth with large cement or amalgam fillings should be carefully inspected, also teeth largely denuded of enamel, dentine or cementum. Heat and cold will reveal hypersensitiveness, and may, by exaggerating the paroxysms of pain disclose the affected tooth. The treatment is radical, either the removal of the pulp or extraction of the tooth. Secondary deposits on the roots of teeth are not frequent causes of pain. They are common in advanced age, especially where there has been considerable disease of the peridental membrane. The exact cause of the disease is not well made out, and it is much a question whether the process in itself is a disease at all. It is not painful, but has by some authorities been supposed to have much to do with those obscure facial neuralgias which can not or have not been traced to other sources. The treatment must be preventive, in relieving all irritable condition of the peridental membrane, or radical extraction of the teeth.

*Reflex irritations* are important factors in medical practice, and more or less attention has been given to these affections by dental practitioners. They are exceedingly difficult to diagnose by the dental practitioner, because an irritation in a very remote part of the body may manifest itself prominently in a dental organ with no evident cause. Diseases of the eye, ear, nose, throat, brain, cord, the thoracic, abdominal and pelvic viscera, to say nothing of many constitutional diseases such as malaria, gout, syphilis, etc., are liable to be the cause of localized pain in and about the dental organs. These reflex irritations of the teeth frequently show themselves in perfectly sound teeth, but more frequently a tooth structurally weak from some cause will be the one selected. The treatment of the affected tooth may give relief, but many reported cases seem to indicate that extraction of the tooth is the only adequate cure. The treatment of these conditions are one of the points at which the dentist and physician should act together. Many serious mistakes have been made because of too much specialization. There is no question but that diseases of the dental organs cause and continue many serious nervous and organic systemic affections in spite of the best medical treatment. But this is not our subject.

There is one cause of toothache that I have been unable to classify, because I do not understand it, and can't find any authority on the subject. As near as I can state it, it is acute irritation without destructive inflammation of the peridental membrane. It generally follows the removal of the pulp by either operative procedures or chemical corrosion and the immediate filling of the root. Especially is it present when irritating chemical germicides or antiseptics have been used to sterilize the root canal. It sometimes follows the treatment and capping of exposed pulps and insertion of fillings. I am not able to briefly outline the symptoms, except to say that there is pain usually without soreness, although soreness may be present. The pain is more or less constant. If from a capped pulp it is sometimes neuralgic. It certainly is irritation of the nerves of the dental tissue surrounding the tooth, but of what nature I am unable to determine. It is exceedingly practical in its bearing on this subject, and is becoming more prominent as the use of rapid destruction and antiseptic treatment of pulp increases. My notion is that it is a secondary effect of the use of irritant anæsthetic or germicidal drugs.

